# Analysis of factors influencing the increase of extracellular water ratio in tumor patients without edema signs

**DOI:** 10.3389/fmed.2025.1642980

**Published:** 2025-08-06

**Authors:** Heling Zhu, Panpan Gan, Hao Jiang, Liangliang Bao, Chengjiang Liu, Jiawen Yu

**Affiliations:** ^1^Department of Oncology, Affiliated Anqing First People’s Hospital of Anhui Medical University, Anqing, China; ^2^Department of General Medicine, Affiliated Anqing First People’s Hospital of Anhui Medical University, Anqing, China; ^3^Department of Clinical Nutrition, Affiliated Anqing First People’s Hospital of Anhui Medical University, Anqing, China

**Keywords:** ECW/TBW, tumor, body composition, phase angle, edema

## Abstract

**Objective:**

This study aims to examine the factors contributing to the increase in extracellular water to total body water ratio (ECW/TBW) among tumor patients without visible edema, and to offer insights into the diagnosis and prompt management of early water retention in such patients.

**Methods:**

A cohort of 274 tumor patients admitted to Anqing First People’s Hospital, affiliated with Anhui Medical University between December 2020 and December 2022, were selected, excluding those with clinically observable edema. General clinical data, main laboratory test outcomes, and body composition indices were gathered. Valuable variables were identified for linear regression analysis using ECW/TBW, based on professional knowledge and univariate analysis results.

**Results:**

Age, gender, hemoglobin, serum sodium, serum potassium, and phase angle (PA) were determined as independent influencing factors for elevated ECW/TBW. ECW/TBW increased with age (partial regression coefficient *B* = 0.009, *p* = 0.001), was higher in males than in females (*B* = −0.349, *p* < 0.001), and was negatively affected by hemoglobin (Hgb) (*B* = −0.003, *p* = 0.039). Serum sodium had a positive influence on ECW/TBW (*B* = 0.019, *p* = 0.011), while serum potassium exhibited a negative effect (*B* = −0.180, *p* = 0.001). PA was the most influential factor among all independent variables affecting ECW/TBW (*B* = −1.006, *p* < 0.001). Nonetheless, Performance Status (PS) score, Patient-Generated Subjective Global Assessment (PG-SGA) score, tumor stage, prealbumin, albumin, and body fat percentage were not identified as independent factors influencing elevated ECW/TBW.

**Conclusion:**

The early factors influencing water retention in tumor patients primarily stem from abnormal cell membrane function, but are also impacted by age, gender, anemia, and electrolyte levels. Timely body composition testing can assist in subsequent intervention decision-making.

## 1 Introduction

Edema is a highly prevalent and burdensome accompanying symptom in tumor patients (1). Previous studies have found that the incidence of tumor-related edema is approximately 5 to 83% (2). As the tumor progresses or malnutrition worsens, a significant majority of patients develop overt edema. Clinically, severe edema manifests not only as limb swelling but can lead to profound functional impairments: it frequently causes difficulties with venous access for infusion, severely limits patient mobility and activities of daily living, and significantly diminishes overall quality of life (3). Furthermore, it predisposes patients to serious complications such as skin breakdown leading to pressure ulcers (bedsores), cellulitis, and deep tissue infections (4). Critically, these issues often necessitate dose reductions, delays, or even discontinuation of essential anti-tumor therapies (e.g., chemotherapy, targeted therapy), thereby potentially compromising treatment efficacy and long-term outcomes. Currently, the primary pathogenic factors of edema in tumor patients are believed to be malnutrition-induced hypoproteinemia, tumor compression-caused disturbance of venous and lymphatic reflux, and venous embolism (5). However, these clinically observable indicators or signs might represent late factors of water retention in the organism, rather than early causes.

Tumor patients with cellular-level functional impairments, metabolic disorders, and electrolyte disturbances can all contribute to increased extracellular water retention. Clinically unobservable water retention might already exist before the onset of hypoproteinemia (6). To quantitatively assess this subclinical fluid accumulation, body composition analysis, particularly the measurement of the extracellular water to total body water ratio (ECW/TBW), has emerged as a valuable tool. An elevated ECW/TBW is a recognized indicator of fluid imbalance and is considered a sensitive marker for detecting early fluid retention, often preceding clinically evident edema (7). This metric offers a significant advantage over traditional clinical assessment (relying on visible edema or pitting) by providing an objective, quantitative measure of fluid distribution shifts that may not yet be apparent on physical examination.

Nevertheless, studies specifically focused on early, subclinical increases in ECW/TBW in tumor patients who do not yet exhibit any visible signs of edema remain scarce, and the clinical implications and underlying factors driving this specific pre-overt-edema state are not fully elucidated. Recent research has further indicated that an elevated extracellular water ratio (ECW/TBW) is a risk factor for disease progression, recurrence, and long-term survival (8, 9), and can mediate early death in tumor patients through other factors (10). This evidence suggests that early detection and intervention targeting fluid imbalance, potentially signaled by elevated ECW/TBW, could help reduce adverse effects and improve patient prognosis.

Therefore, to address this gap and explore the potential for early identification, this study employs body composition analysis via BIA. We specifically aim to assess the ECW/TBW ratio in tumor patients who present without visible signs of edema. Our primary objective is to evaluate the prevalence of an abnormally elevated ECW/TBW in this population and identify potential demographic, clinical, tumor-related, and nutritional factors associated with this early indicator of fluid dysregulation. By elucidating these factors, this study seeks to provide valuable insights for the early recognition of subclinical fluid retention and inform strategies for timely clinical intervention aimed at preventing or delaying the onset of overt, debilitating edema and its associated complications in tumor patients.

## 2 Materials and methods

### 2.1 Study design and participants

This study used the retrospective analysis method, and patients with solid tumors admitted to Anqing First People’s Hospital Affiliated to Anhui Medical University from December 2020 to December 2022 were collected. This study was approved by the Ethics Committee of the Affiliated Anqing First People’s Hospital of Anhui Medical University. The ethical number is AQYY-YXLL-KJXM-10. The trial enrolled patients age >18 years who had pathologically diagnosed solid malignant tumors and able to stand and undergo body composition analysis testing, and provided written informed consent to participate in the study. And patients with the following features were excluded: with double or multiple primary tumors; severe liver or kidney diseases; cardiac function grade III or above; lower extremity venous thrombosis; clinically observable peripheral edema and severe plasma cavity effusion. Four tumor patients without biochemical examination, four cases of central nervous system tumors, 13 cases of tumors with multiple primary or unknown origin, one case of soft tissue tumors of unknown stage, three cases of liver cirrhosis, and seven cases of chronic cardiac and renal insufficiency were excluded, leaving 274 tumor patients.

### 2.2 Methods for the measurement of body composition indicators including ECW/TBW

After patients fasted or stopped eating for 2 h and emptied urine and feces, body composition indices such as extracellular water/total body water (ECW/TBW), skeletal muscle mass index (SMI), body fat percentage (BFP), phase angle (PA), and body mass index (BMI) were measured using the Inbody770 body composition analyzer.

### 2.3 General clinical data collection and experimental index measurement methods

General clinical data such as age, gender, tumor stage, tumor type, treatment history within 1 month and PS score were collected through history taking, and the nutritional status of patients was evaluated based on the Patient-Generated Subjective Global Assessment (PG-SGA) score. Hemoglobin (Hgb), prealbumin (PAB), albumin (ALB), and electrolytes were measured using the Sysmex XN-3100 automated hematology analyzer and the Hitachi 7600 automatic biochemical analyzer.

### 2.4 Statistical methods

SPSS25 software was employed for statistical analysis. For the collected measurement data, normality analysis was performed, Spearman correlation test was utilized for univariate correlation analysis, and rank sum test was employed for comparison of rank data. Indicators with statistically significant correlation analysis were included in the linear regression analysis (stepwise regression). The test level was set at *α* = 0.05.

## 3 Results

### 3.1 General data analysis

According to the established inclusion and exclusion criteria, a total of 274 patients met the criteria for this study. The tumor types included esophageal cancer (*n* = 42), gastric cancer (*n* = 34), colorectal cancer (*n* = 30), lung cancer (*n* = 29), breast cancer (*n* = 51), head and neck tumor (*n* = 21), gynecological and reproductive system tumor (*n* = 34), urinary system tumor (*n* = 7), pancreatic cancer (*n* = 8), hepatobiliary tumor (*n* = 10), and soft tissue tumor (*n* = 8). The treatments within 1 month included no anti-cancer treatment (*n* = 110), non-platinum chemotherapy (including combined targeted or immunotherapy, etc.) (*n* = 53), platinum-based chemotherapy (including combined targeted or immunotherapy, etc.) (*n* = 49), and others treatment (including targeted, immunological, surgery, endocrinology but excluding chemotherapy) (*n* = 62). For additional patient details, refer to Table 1.

**Table 1:** Baseline characteristics of patients (*N* = 274).

Clinical data		People tested (*N* = 274)		
		*N* (%)	*x̄* ± s	Median
Age*	21 ~ 88		60.00 ± 10.55	58
Gender	Male	144 (52.55)		
	Female	130 (47.45)		
PS score	0	47 (17.15)		
	1	206 (75.18)		
	2	21 (7.66)		
PG-SGA score*	1–19			4
Tumor type	Esophagus cancer	42 (15.33)		
	Gastric cancer	34 (12.41)		
	Colorectal cancer	30 (10.95)		
	Lung cancer	29 (10.58)		
	Breast cancer	51 (18.61)		
	Head–neck cancer	21 (7.67)		
	Gynecologic cancer	34 (12.41)		
	Pancreatic cancer	8 (2.92)		
	Urologic cancer	7 (2.55)		
	Soft connective tissue cancer	8 (2.92)		
	Hepatobiliary cancer	10 (2.74)		
Tumor stage	Stage I	26 (9.49)		
	Stage II	62 (22.63)		
	Stage III	77 (28.10)		
	Stage IV	109 (39.78)		
Treat	No anti-cancer treatment	110 (40.15)		
	Non-platinum treatment	53 (19.34)		
	Platinum-based treatment	49 (17.88)		
	Others treatment	62 (22.63)		
Hgb (g/L)	67.00 ~ 163.00		110.43 ± 16.93	
PAB (mg/L)	44.00 ~ 495.00		229.65 ± 76.45	
ALB (g/L)	27.1 ~ 49.2		39.16 ± 4.62	
Serum sodium (mmol/L)*	121.8 ~ 153.00			141.10
Serumpotassium (mmol/L)*	2.83 ~ 5.85			4.21
ECW/TBW (%)*	37.30 ~ 42.00			39.10
SMI (kg/m^2^)*	3.20 ~ 9.70			6.50
BFP (%)*	3.00 ~ 44.60			24.10
PA (°)*	2.20 ~ 6.60			4.60
BMI (kg/m^2^)	12.90 ~ 32.80		21.75 ± 3.55	

“*” denotes non-normally distributed variable conforming to the KS test; “PS” is Performance Status; “PG-SGA” is Patient-Generated Subjective Global Assessment; “Hgb” is hemoglobin; “PAB” is prealbumin; “ALB” is albumin; “ECW/TBW” is the extracellular water ratio; “SMI” is skeletal muscle mass index; “BFP” is body fat percentage; “PA” is phase angle; and “BMI” is body mass index.

### 3.2 Univariate analysis of clinical characteristics and laboratory indices with ECW/TBW

ECW/TBW was positively correlated with age and PG-SGA score, and negatively correlated with Hgb, PAB, ALB, and serum potassium levels. Male patients exhibited higher ECW/TBW than female patients, and patients with high PS scores and later tumor stages had higher ECW/TBW than those with lower PS scores and earlier tumor stages. Significant heterogeneity in baseline ECW/TBW ratio was observed across the different tumor types included in the study. Specifically, post-hoc pairwise comparisons revealed that patients with esophageal cancer had significantly higher ECW/TBW ratios compared to those with breast cancer. Similarly, patients with gastric cancer exhibited higher ECW/TBW ratios than both breast cancer patients and gynecological cancer patients. The no anti-cancer treatment, non-platinum chemotherapy, and platinum-based chemotherapy group exhibited higher ECW/TBW than the others treatment group. No statistically significant correlation was found between serum sodium levels and ECW/TBW (see Table 2).

**Table 2:** Univariate analysis of clinical characteristics and laboratory indices with ECW/TBW (*N* = 274).

Clinical data	ECW/TBW (%)			
	*r_s_*	*p*	Rank average	*p*
Age	0.497	<0.001		
Gender			Male: 155.90	<0.001
			Female: 117.12	
PS score			0: 105.77	<0.001
			1: 139.75	
			2: 186.48	
PG-SGA score	0.453	<0.001		
Tumor type			Esophagus cancer^a^: 170.45	<0.001
			Gastric cancer^b^: 179.29	
			Colorectal cancer: 140.23	
			Lung cancer: 153.38	
			Breast cancer^c^: 82.88	
			Head–neck cancer: 159.19	
			Gynecologic cancer^d^: 100.34	
			Pancreatic cancer: 161.69	
			Urologic cancer: 181.29	
			Soft connective tissue cancer: 141.31	
			Hepatobiliary cancer: 109.05	
Tumor stage			Stage I: 94.85	<0.001
			Stage II: 118.32	
			Stage III: 133.24	
			Stage IV: 161.59	
Treat			No anti-cancer treatment^e^: 153.33	<0.001
			Non-platinum treatment^f^: 139.25	
			Platinum-based treatment^g^: 154.32	
			Others treatment^h^: 94.14	
Hgb (g/L)	−0.424	<0.001		
PAB (mg/L)	−0.463	<0.001		
ALB (g/L)	−0.443	<0.001		
Serum sodium (mmol/L)	0.051	0.399		
Serum potassium (mmol/L)	−0.282	<0.001		

“*r_s_*” represents the correlation coefficient of the Spearman test; “ECW/TBW” stands for extracellular water/total body water; “PS” denotes performance status; “PG-SGA” refers to patient-generated subjective global assessment; “Hgb” is hemoglobin; “PAB” is prealbumin; “ALB” is albumin; *p*(a vs. c)/*p*(b vs. c)/*p*(b vs. d)/*p*(e vs. h)/*p*(f vs. h)/*p*(g vs. h) < 0.05. ^a-d^ represent the average value of ECW/TBW of patients with esophageal cancer, gastric cancer, beer cancer and gynecological cancer respectively; ^e-h^ represents the average ECW/TBW of patients without anti-cancer treatment, non-platinum treatment, platinum-containing treatment and other treatment methods respectively.

### 3.3 Univariate analysis between each human component index

ECW/TBW was negatively correlated with BFP, PA, and BMI, and showed no correlation with SMI. SMI was negatively correlated with BFP, but positively correlated with PA and BMI. BFP demonstrated a weak positive correlation with PA and a strong positive correlation with BMI (correlation coefficient *r_s_* = 0.754); however, PA was positively correlated with BMI (see Table 3).

**Table 3:** Univariate analysis of clinical characteristics and laboratory indices with ECW/TBW (*N* = 274).

Indices	ECW/TBW		SMI		BFP		PA	
	*r_s_*	*p*	*r_s_*	*p*	*r_s_*	*p*	*r_s_*	*p*
SMI	−0.027	0.660	–	–	–	–	–	–
BFP	−0.280	<0.001	−0.124	0.041	–	–	–	–
PA	−0.896	<0.001	0.310	<0.001	0.185	0.002	–	–
BMI	−0.303	<0.001	0.471	<0.001	0.754	<0.001	0.399	<0.001

“*r_s_*” represents the correlation coefficient of the Spearman test; “ECW/TBW” stands for extracellular water/total body water; “SMI” denotes skeletal muscle mass index; “BFP” refers to body fat percentage; “PA” is phase angle; “BMI” is body mass index.

### 3.4 Linear regression analysis of clinical characteristics and indicators with ECW/TBW

A strong correlation between BMI and BFP was found, and multiple covariance was diagnosed by covariance. Based on expertise consideration in the alteration of body composition, the specificity of BMI for the dependent variable in this study was not sufficient, so it was not included in the regression analysis. Serum sodium, an important factor affecting osmotic pressure, may be related to the change in extracellular water. Although there was no statistically significant difference in univariate analysis, it was still included in the analysis based on professional knowledge and research purpose. Other indicators that were statistically significant for univariate analysis were included in the analyses. The results showed that age, gender, Hgb, serum sodium, serum potassium, and PA were all factors affecting ECW/TBW. Serum sodium, which did not show a statistical difference in univariate analysis, exhibited statistical significance in regression analysis (*B* = 0.019, *p* = 0.011). The largest absolute value of the standardized partial regression coefficient was found for PA (Beta = −0.779). PS score, PG-SGA score, tumor stage, PAB, ALB, and BFP did not affect ECW/TBW. The regression equation test statistic *F* = 235.577, *p* < 0.001; *R*^2^ = 0.841, adjusted *R*^2^ = 0.838 (see Table 4).

**Table 4:** Multiple linear regression analysis of ECW/TBW and influencing factors (*N* = 274).

Independent variable	*B*	SE	Beta	95%CI	*t*	*p*
Constant term	47.746	1.036		39.707 ~ 43.785	40.313	<0.001
Age	0.008	0.003	0.094	0.003 ~ 0.013	3.291	0.001
Gender	−0.361	0.050	−0.192	−0.460 ~ −0.261	−7.147	<0.001
Hgb (g/L)	−0.004	0.002	−0.075	−0.007 ~ 0.001	−2.712	0.007
Serum sodium (mmol/L)	0.021	0.007	0.074	0.007 ~ 0.034	2.950	0.003
Serum potassium (mmol/L)	−0.190	0.053	−0.094	−0.295 ~ −0.085	−3.564	<0.001
PA (°)	−1.002	0.038	−0.776	−1.077 ~ −0.928	−26.391	<0.001

*F* = 235.577, *p* < 0.001; *R*^2^ = 0.841, adjusted *R*^2^ = 0.838. “*B*” represents the unstandardized partial regression coefficient; “Beta” denotes the standardized partial regression coefficient. “CI” refers to the confidence interval; “PS” is performance status; “PG-SGA” is patient-generated subjective global assessment; “Hgb” is hemoglobin; “PAB” is prealbumin; “ALB” is albumin; “BFP” is body fat percentage; “PA” is the phase angle.

## 4 Discussion

### 4.1 Explanation of ECW/TBW univariate analysis results

An approximately 50–60% of the human body is composed of water, which includes intracellular water (ICW) and extracellular water (ECW). The ratio of ICW to ECW is approximately 2:1, with ECW mainly composed of plasma and tissue fluid in a ratio of around 1:3 (11). Generally, total plasma volume remains relatively constant, and edema (12) mainly results from an imbalance in intra- and extra-vascular fluid exchange or from water and sodium retention in the body. This imbalance is associated with increased tissue fluid in the extracellular fluid and excessive accumulation in subcutaneous tissue.

The univariate analysis of this project suggested that ECW/TBW was associated with age and tumor stage. ECW/TBW was higher in male than in female patients and increased with PS score and PG-SGA score. ECW/TBW was negatively associated with laboratory indices such as Hgb, PAB, and ALB. Regarding body composition analysis indices, ECW/TBW was negatively associated with BFP, PA, and BMI, but showed no associated with SMI. These results indicate that factors associated with the increase of ECW/TBW in tumor patients are complex, and the extracellular water ratio in tumor patients may rise with increasing age, disease progression, decreasing functional ability, worsening malnutrition, decreasing body fat content, weight loss, lowering protein levels, and aggravating anemia. While previous studies (13–15) reported a correlation between SMI and ECW/TBW, our data revealed no such association. We propose that early fluid accumulation primarily impacts muscle quality before detectable mass loss occurs. This hypothesis aligns with evidence on myosteatosis. In advanced hepatocellular carcinoma patients receiving immunotherapy, myosteatosis independently predicts reduced survival (16), indicating its role in driving metabolic dysfunction and inflammation, which may promote ECW expansion prior to SMI decline. Notably, only specific myosteatosis cutoffs significantly correlate with survival in oncology cohorts (17), underscoring that muscle quality alterations are key early indicators of fluid imbalance. Phase angle (PA), a bioimpedance-derived marker of cellular integrity, further bridges this gap. In hip fracture rehabilitation, ECW/TBW improvement is closely linked to PA elevation and precedes SMI recovery (18). This explains why muscle strength declines with ECW/TBW elevation even without SMI changes (19). Thus, SMI may lack sensitivity to early fluid-related pathology, warranting prioritization of muscle quality metrics (e.g., PA, myosteatosis) in early-stage monitoring.

Since patients with obvious edema were not included in this study, it is hypothesized that early accumulation of extracellular fluid may primarily impact muscle function without being associated with skeletal muscle loss, potentially explain the lack of statistical correlation between SMI and ECW/TBW in this study. Skeletal muscle loss may be more prevalent in patients with poorer nutritional status and more severe water retention. This finding also suggests that SMI may not be a sensitive indicator of early water retention in oncology patients, and this parameter was not included in the subsequent regression analysis.

PA is an indicator derived from the bioelectrical impedance principle to assist in diagnosing nutritional status, which can specifically reflects the state of structural integrity and functional impairment of human cell membranes (20). Low PA can be diagnosed when PA is below 5.0° in males and 4.6° in females (21). In our cohort, 72% of males and 39% of females exhibited PA values below these sex-specific thresholds. This high prevalence of subthreshold PA—particularly among males—aligns with our observed median PA (4.5° in males, 4.6° in females) and provides essential context for the robust predictive power of PA in our regression models for ECW/TCW. The marked sex disparity in malnutrition risk warrants further investigation but may reflect differences in body composition or disease severity profiles. A decrease in PA indicates poor prognosis, increased complications, and shortened survival (22, 23). This study’s results revealed a strong negative correlation between PA and ECW/TBW (correlation coefficient *r_s_* = −0.896, *p* < 0.001). Consequently, it can be inferred that the mechanisms of extracellular water ratio increase in early and late tumor stages may significantly differ. The structural damage and functional impairment of cell membranes in tumor patients may be a key factor associated with the increase in extracellular water in the early stages. Paying attention to the early increase of ECW/TBW may allow for earlier prediction of poor prognosis in patients. However, both basic and clinical studies in this area are lacking and require further reporting.

Malignancies originating from different organs exhibit distinct biological behaviors, metabolic consequences, and treatment paradigms, which could theoretically confound associations involving body composition parameters like ECW/TBW (24, 25). Univariate analysis confirmed significant differences in ECW/TBW across tumor types (*p* < 0.05). Esophageal cancers (*n* = 42) showed higher ECW/TBW vs. breast cancers (*n* = 51) (*p* = 0.002); gastric cancers (*n* = 34) showed higher ECW/TBW vs. breast cancers (*n* = 51) (*p* < 0.001); gastric cancers (*n* = 34) had higher ECW/TBW vs. gynecological cancers (*n* = 34) (*p* = 0.011). However, we note that the observed inter-tumoral differences in ECW/TBW ratios may be largely associated to the uneven distribution of disease stages and variations in nutritional status across patient cohorts in this study: Stage Distribution: The proportion of early-stage (I–II) patients was significantly higher in the breast cancer (58.8%) and gynecological cancer (49.0%) groups compared to the esophageal (35.7%) and gastric cancer (26.5%) groups. Conversely, advanced-stage (III–IV) disease predominated in the esophageal (64.3%) and gastric cancer cohorts (73.5%), substantially exceeding rates in the breast (41.2%) and gynecological cancer groups (51.0%). Nutritional Status: PG-SGA Score: Mean PG-SGA scores were significantly elevated in the esophageal (7.07) and gastric cancer groups (6.35) versus the breast (3.03) and gynecological cancer groups (1.57), indicating a higher burden of malnutrition in the former. PA: The prevalence of patients with PA <4.6° was markedly greater in the esophageal (59.5%) and gastric cancer groups (55.9%) than in the breast (23.5%) and gynecological cancer groups (23.5%). Future research employing larger, tumor-specific cohorts is essential to validate and refine our observations within homogeneous populations and to explore potential nuances in the relationship between ECW/TBW and body composition alterations to different malignancies.

The potential confounding effect of concurrent anti-cancer treatments on body composition parameters, particularly fluid balance (reflected by ECW/TBW). Treatments such as chemotherapy (especially platinum-based), immunotherapy, targeted therapies can influence hydration status, renal function, electrolyte levels, thereby potentially impacting BIA-derived measures like PhA and ECW/TBW (26, 27). Our retrospective analysis, incorporating treatment data received within 1 month prior to BIA, confirmed this concern at the univariate level. We observed significantly higher ECW/TBW values in patients receiving no anti-cancer treatment, platinum-based chemotherapy, or non-platinum chemotherapy compared to those receiving other treatment. This might be associated to the fact that effective treatment reversed the upward trend of ECW/TBW caused by the tumor itself, keeping it at a relatively low level. Moreover, based on further analysis of the data, we found that these differences are closely related to the concurrent changes in nutritional and metabolic conditions. The PG-SGA scores and PA of the other treatment groups were better than those of the no anti-cancer treatment and platinum-based chemotherapy groups. Future prospective studies investigating body composition in cancer patients should prioritize the systematic, detailed prospective collection of comprehensive anti-cancer treatment data and treatment-related toxicities. This will allow for more robust adjustment and the exploration of specific treatment effects on body composition parameters.

### 4.2 Explanation of the results of regression analysis

The extracellular water ratio in tumor patients generally increases with disease progression, heightened malnutrition, and decreased protein levels (6), which is consistent with findings from previous studies. However, the linear regression analysis in this study revealed that malnutrition, prealbumin, and albumin were not independently associated with the elevated ECW/TBW in tumor patients.

Serum sodium, the most critical electrolyte for maintaining tissue osmotic pressure, did not exhibit a significant correlation with the extracellular water ratio in the univariate analysis of this project. Nevertheless, based on professional knowledge, the research team decided to include serum sodium levels in the regression analysis. The results indicate that serum sodium levels are independently associated with the increase in the extracellular water ratio. Furthermore, age, gender, potassium, and hemoglobin, which are not classically considered to be associated with edema, also demonstrated independent associations.

Previous results from physical examinations in healthy populations revealed that the extracellular water ratio was lower in males than in females, and it gradually increases with age (28). The regression analysis results in this study showed that although the partial regression coefficient of age to ECW/TBW was minimal, it still exhibited statistical significance. This finding indicates that the change of ECW/TBW in tumor patients with age was generally consistent with that of normal individuals. Notably, the discovery that the extracellular water ratio is higher in males than in females diverges from the water distribution characteristics of the normal population. This discrepancy may be due to differences in the regulatory effects of androgens and estrogens on the human renin-angiotensin system (RAS), potentially resulting in higher RAS activity in males than in females. This higher activity is evidenced by a greater tendency for males to develop water sodium retention, hypokalemia, and hypertension compared to females (29). Many tumors exhibit increased expression of angiotensin and its receptors (30, 31), and RAS is relatively activated in some tumor populations. The positive regulation of this system by androgens may contribute to the higher ECW/TBW in males compared to females observed in this study. The association of electrolytes on ECW/TBW in the regression analysis shows that an increase in serum sodium and a decrease in serum potassium accompany an increase in the extracellular water ratio, further supporting this hypothesis. RAS activation in tumor patients is a current research hotspot. This system activation can promote tumor progression and metastasis through downstream signaling pathways, leading to poorer patient survival. For example, Prof. Ryan M. Young’s team found that oncogenic RAS mutations activate mTORC1 signalling in multiple myeloma and combining mTORC1 and MEK/ERK inhibitors synergize to improve survival in preclinical models (32). Meanwhile, angiotensin-converting enzyme inhibitors, which inhibit the activity of this system, can improve the survival of some tumors. Consequently, patients with increased ECW/TBW, combined with high serum sodium and low potassium, should be monitored closely.

The results of the regression analysis suggested that a decrease in hemoglobin was also associated with an increase in the ratio of extracellular water. The phase angle analysis results were consistent with the univariate analysis and still exhibited the strongest association with the extracellular water ratio (standardized partial regression coefficient Beta = −0.779, *p* < 0.001). Therefore, it can be speculated that the early increase in ECW/TBW may primarily stem from abnormal cell membrane function and is not due to factors such as hypoproteinemia, increased microvascular permeability, and other imbalances in intra- and extra-vascular fluid exchange. The modes of water entering and leaving the cell include free diffusion and assisted diffusion, with the direction of movement depending on the osmotic pressure inside and outside the cell membrane.

Aquaporin (AQP) is a carrier protein that facilitates the diffusion of water into and out of cell membranes, and is widely expressed in various tissues and organs throughout the body. Its dysregulation has been observed in a variety of tumors (33, 34). The regulation of its expression is influenced by tissue hyperosmolarity, hypoxia, and ischemia. Anemia can exacerbate tissue hypoxia, which can further inducing dysregulation of water channel protein expression and result in the accumulation of hyperosmotic metabolic products in the body’s tissue fluid (35). This facilitates the rapid movement of water across cell membranes along the osmotic pressure gradient. Recent studies suggest (36) that edema may also promote tumor proliferation by inducing abnormal expression of AQP. The role of aquaporins in tumor progression and metastasis is a current research hotspot, but some aspects of the mechanism remain unclear and warrant further investigation through clinical and basic research. Relevant targeted drugs are also undergoing active trials (37, 38).

The integrity of the structure and function of the lipid bilayer and membrane proteins in human cell membranes is the physiological basis for normal phase angle (PA). A reduction in PA indicates a disruption of the cell membrane structure that maintains the homeostasis of the internal environment, which may also facilitate, to some extent, the passage of water through the cell membrane by free diffusion.

Based on the above analysis, it is preliminarily proposed that the early elevation of ECW/TBW is primarily linked to cell membrane damage in tumor patients. The combination of relative activation of the RAS system, associated with water-sodium retention and potassium excretion, along with tissue hypoxia related to anemia, promotes increased tissue osmotic pressure in the body. This may be accompanied by abnormal expression of water channel proteins, ultimately correlating with the increased transfer of intracellular water to the extracellular compartment. Age may be a physiological factor correlated with the decline of cell membrane function, and differences in the degree of RAS regulation by sex hormones could be linked to increased extracellular water more commonly in males. The early increase in ECW/TBW, with the risk of adverse reactions and disease recurrence progression, also warrants follow-up study.

The present study has several limitations. First, the retrospective single-center design is prone to bias. Second, the small sample size may also be a source of bias. Third, further prospective studies are needed to confirm whether the ECW/TBW predicts long-term OS, or whether it exhibits dynamic changes according to the patient’s general condition and treatment response. Previous studies have indicated associations between the ECW/TBW and clinical outcomes in cases involving chronic liver diseases, renal disorders, heart failure (39–41). Kaiwen Zheng et al. (6) found an association between ECW/TBW and the prognosis of people with advanced cancer. However, because they were selected from patients with advanced tumors and did not remove cases with obvious clinical signs of edema, they could not be corrected in time with appropriate clinical interventions. We will continue to follow up in later studies to include more samples and develop more reliable statistical models to examine the associations of tumor type, treatment strategy, specific chemotherapy regimen, and other factors on the increase in extracellular water, as well as incorporate patient follow-up data to track the occurrence of edema and its potential association with patient survival.

In conclusion, this study uniquely identifies early elevation of the extracellular water ratio (ECW/TBW) in patients with subclinical edema, independent of common drivers like disease progression or malnutrition. This underscores the need for vigilance in high-risk subgroups—particularly older males with anemia, hypernatremia, and/or hypokalemia—where body composition assessment can detect subclinical fluid shifts and associated abnormalities (e.g., low phase angle). Timely intervention targeting these modifiable factors may help maintain homeostasis and potentially mitigate complications and cancer progression.

## Data Availability

The raw data supporting the conclusions of this article will be made available by the authors, without undue reservation.
